# A novel necroptosis-related lncRNA signature for predicting prognosis and anti-cancer treatment response in endometrial cancer

**DOI:** 10.3389/fimmu.2022.1018544

**Published:** 2022-11-16

**Authors:** Wei-Peng He, Yu-Ying Chen, Lin-Xiang Wu, Yun-Yun Guo, Ze-Shan You, Guo-Fen Yang

**Affiliations:** Department of Gynecology, The First Affiliated Hospital, Sun Yat-Sen University, Guangzhou, China

**Keywords:** lncRNAs, necroptosis, endometrial cancer, immune infiltration, prognosis

## Abstract

**Background:**

Necroptosis, a form of programmed cell death, underlies tumorigenesis and the progression of cancers. Anti-cancer strategies targeting necroptosis have increasingly been shown to present a potential cancer therapy. However, the predictive utility and anticancer sensitivity value of necroptosis-related lncRNAs (NRLs) for endometrial cancer (EC) are currently unknown.

**Methods:**

EC patient gene expression profiles and the corresponding clinical information collected from The Cancer Genome Atlas were used to identify NRLs that constituted a predictive signature for EC. The functional pathways, immune status, clinicopathological correlation, and anticancer drug sensitivity of the patients relative to the NRLs signatures were analyzed.

**Results:**

A signature composed of 7 NRLs (AC019080.5, BOLA3-AS1, AC022144.1, AP000345.2, LEF1-AS1, AC010503.4, and RPARP-AS1) was identified. The high-risk patient group with this signature exhibited a poorer prognosis and lower survival rate than low-risk group lacking this signature. This necroptosis-related lncRNA signature had a higher predictive accuracy compared with other clinicopathological variables (area under the receiver operating characteristic curve of the risk score: 0.717). Additionally, when patients were stratified based on other clinicopathological variables, the overall survival was significantly shorter in the high-risk versus low-risk group across all cohorts. Gene set enrichment analysis (GSEA) revealed that immune- and tumor-related signaling pathways and biological processes were enriched in the high-risk group compared to the low-risk group. Single-sample gene set enrichment analysis (ssGSEA) additionally showed that the resulting risk score was strongly correlated with EC patient immune status. Finally, patients with high-risk scores were more sensitive to the anti-cancer drugs such as Docetaxel, Mitomycin.C, Vinblastine, AZD.2281 (olaparib), AZD6244, and PD.0332991 (Palbociclib).

**Conclusion:**

These findings reveal a novel necroptosis-related lncRNA signature for predicting EC patient prognosis and shed new light on anticancer therapy strategies for EC.

This manuscript has been thoroughly edited by a native English speaker from an editing company. Editing Certificate will be provided upon request.

## Introduction

Endometrial cancer (EC) is one of the most common malignancies of female genitalia worldwide (1). Based on clinical and hormonal features, EC is classified into two primary types that have dramatically different prognoses: oestrogen-dependent Type I and oestrogen-independent Type II (1, 2). The more prevalent Type I EC is typically endometrioid carcinoma, low grade with a favorable prognosis, whereas Type II tumors are high grade, and can have an adverse prognosis with a highly recurrent tendency even in the early stages (2, 3). The molecular features of EC are crossed according to the above classification, and some cases are not completely consistent with the pathological features. For example, this classification system for high-grade subtypes is limited in its reproducibility, in particular the distinction between high-grade endometrioid and serous carcinomas (4, 5). Based on the molecular genome sequence analysis, EC has recently been divided into four subgroups: microsatellite instability (MSI), POLE ultra-mutated, copy-number high and copy-number low (3). Despite recent investigations of therapies targeting immune checkpoint inhibitors and immune-related pathways, the response rates of these treatments remain low (6). As such, there is an urgent need to identify reliable predictive biomarkers and targeted therapeutic strategies for EC.

Necroptosis is a form of programmed inflammatory cell death (7), mediated by MLKL, RIPK3, RIPK1, CYLD, cIAP1/2, and caspase-8 and inhibited by GSK843, GSK872, Nec-1, NSA, zVAD-fmk smac and other mimetics (7, 8). Recent studies have shown that necroptosis plays a pivotal role in the progression, metastasis, immunosurveillance, and prognosis of cancer (8–11). However, other research suggests that necroptosis may prevent tumor progression (12, 13). Necroptosis has become a hot topic in cancer therapy in recent years. For instance, RIPK1 was identified as a therapeutic target in pancreatic cancer (14). CBL0137 was shown to benefit immune checkpoint blockade-based therapies (15). Moreover, necroptosis-related signatures have been found to act as prognostic biomarkers for several cancers (16, 17).

Long non-coding RNAs (lncRNAs) are noncoding RNAs longer than 200 nucleotides that lack protein coding potential and regulate tumorigenesis and proliferation (18, 19). For example, LncRNA OIP5-AS1 regulates the proliferation and invasion of EC cell *via* controlling the PTEN/AKT pathway by targeting miR-200c-3p (20). LncRNA NEAT1 enhances the migration, invasion and proliferation of EC cells by targeting the miR-144-3p/EZH2 axis (21). LncRNA DCST1-AS1 promotes EC progression *via* regulating the MiR-873-5p/CADM1 and MiR-665/HOXB5 pathways (22). LncRNA MEG3 inhibits EC tumorigenesis and progression *via* the PI3K pathway (23). LncRNA LINC00672 promotes EC chemosensitivity and impacts EC malignancies by regulating LASP1 expression (24). Given the key role of lncRNA in tumor development, lncRNAs-related prognostic signatures of EC patients have been extensively explored (25–29). However, the roles and mechanisms of necroptosis-related lncRNAs (NRLs) in EC remain largely unknown.

With the rapid advances of The Cancer Genome Atlas (TCGA), big data mining is emerging as a promising method to explore the tumorigenesis mechanisms, related prognostic markers, and cancer therapeutic targets. Here, we constructed a new necroptosis-related lncRNA signature to predict the prognosis of EC and reveal anti-cancer sensitivity in EC patients. The goal of this work was to provide guidance for clinical diagnosis and treatment.

## Materials and methods

### Data collection and preprocessing

RNA-sequencing data were acquired from TCGA database (https://portal.gdc.cancer.gov/), consisting of 23 normal specimens and 554 EC specimens (accessed on April 23, 2022). The data were downloaded as fragments per kilobase million (FPKM). The clinical information of these EC patients was also downloaded. Patients with adverse overall survival (OS) less than 30 days or without OS values were excluded. A total of 205 necroptosis-related genes were obtained from the Kyoto Encyclopedia of Genes and Genomes (KEGG) website (https://www.kegg.jp/) and previously reported literature (30). A total of 178 necroptosis-related genes were retrieved from the mRNA expression matrix of EC in TCGA database (**Supplementary Table S1**).

### Differentially expressed necroptosis-related genes and functional enrichment analysis

Necroptosis-related differentially expressed genes (DEGs) were selected based on a screening criterion of |log 2 fold change (FC) > 1| and a false discovery rate (FDR)<0.05 using the “limma” package. KEGG and Gene Ontology (GO) analyses were performed using the “clusterProfiler” R package.

### Establishment and verification of the necroptosis-related lncRNA prognostic signature

A total of 539 NRLs were obtained by Pearson’s correlation analysis according to a screening criteria of Pearson correlation coefficient |R|>0.3 and p<0.001. We used the “survival” R package to select prognostic necroptosis-related lncRNAs with p< 0.05 by univariate Cox regression analysis. (**Supplementary Table S2**). We then performed least absolute shrinkage and selection operator (LASSO) regression analysis to filter NRLs using 10-fold cross-validation and multivariate Cox regression analysis was ultimately used to obtain the risk models (**Supplementary Table S3**). The risk score was calculated as follows:


$$\text{Risk score}=\sum_{\text{i}=1}^{\text{n}}\text{Coef}\left(\text{i}\right)\times \text{x}\left(\text{i}\right)$$

in which x(i) and Coef (i) represent the expression levels of each NRLs and the regression coefficient, respectively. We divided EC patients into low- and high-risk groups according to the median risk score. Kaplan Meier (K-M) survival curves conducted using the “survival” R package were utilized to evaluate the differences of the overall survival (OS) between the low- and high-risk groups. Receiver operating characteristic (ROC) curves built using the “timeROC” R package were utilized to evaluate the predictive accuracy of the NRLs signature. The relationships between clinicopathological parameters and risk score were also assessed.

### Coexpression network analysis and principal component analysis

An mRNA-lncRNA coexpression network between 7 NRLs and their corresponding necroptosis-related genes was constructed using the Cytoscape software (Version 3.6.1). We additionally created a Sankey diagram to verify the correlation between NRLs and the corresponding necroptosis-associated genes using the “ggalluvial” R package. Using the “scatterplot3d” R package, principal component analysis (PCA) was utilized to decrease the dimensionality for visualization purpose.

### Construction and calibration of nomogram

We constructed a nomogram based on the risk score and the clinicopathological variables of age, stage, and grade using the “rms” R package. This was used to predict the 1-, 3-, and 5-year OS of EC patients. Additionally, we created a calibration curve to validate the predictive efficacy of the nomogram model.

### Gene set enrichment analysis

GSEA software (version 4.2.3, http://www.gsea-msigdb.org/) was used to identify the significantly enriched pathways between the high- and low-risk groups (31). Here, the GO assays were performed to analyze biological functions, while KEGG assays were conducted to analyze signaling pathways. Results with FDR<0.25 and p<0.05 were considered statistically significant.

### Analyses of immune checkpoint, immune cell infiltration, and immunotherapy

The differences in immunological functions, immune cell infiltration scores, and pathways between low- and high-risk groups were analyzed using single-sample gene set enrichment analysis (ssGSEA) using the “GSEABase” and “GSVA” R packages. We also compared the expression levels of immunological checkpoints between low- and high-risk sets and explored the relationship between the risk score and the clinical PD-L1 subtypes.

### Prediction of drug sensitivity in the risk model

To predict each group’s potential response to common chemotherapy drugs, we calculated the half-maximal inhibitory concentration (IC50) of compounds in EC patients using the “pRRophetic” R package. The compounds were obtained in the Genomics of Drug Sensitivity in Cancer (GDSC) database. The differences in IC50 values between the low- and high-risk subsets were evaluated using Wilcoxon signed-rank test.

### Statistical analysis

R software (Version 4.1.2) was applied to carry out all statistical analyses. The Wilcoxon test was utilized to assess the differences between the low- and high-risk groups. The Log-rank test and Cox proportional hazards model were utilized to compare the differences in survival between the two groups. The correlations between the two sets were explored by Spearman’s correlation analysis. The “survivalROC” R package was used to depict the ROC curves and calculate the values of the area under the curve (AUC). The “GSVA” R package was used in ssGSEA. P< 0:05 was considered statistically significant.

## Results

### Functional enrichment analysis of necroptosis-related genes

**Figure 1:**
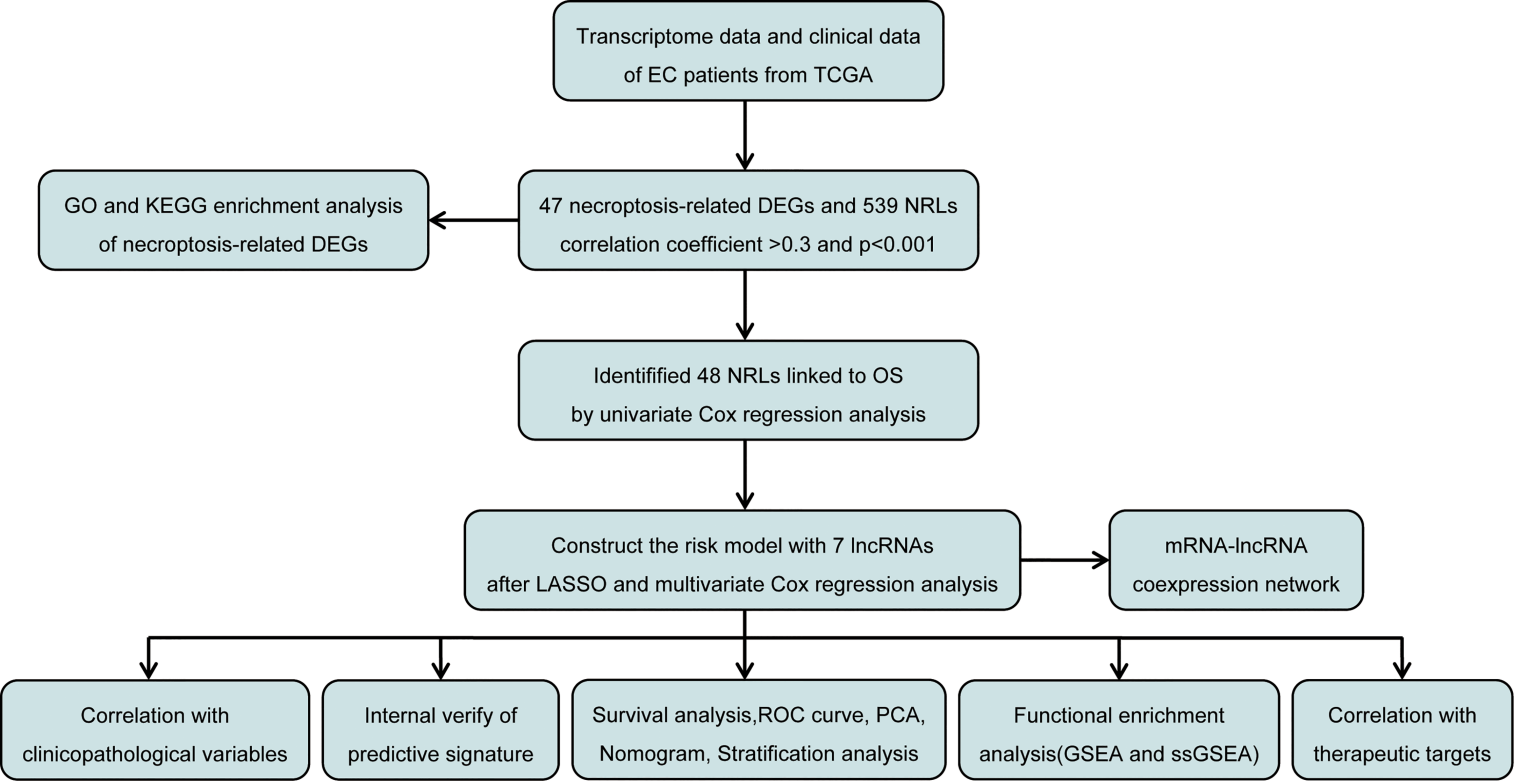
Flow diagram of the study design.

**Figure 2:**
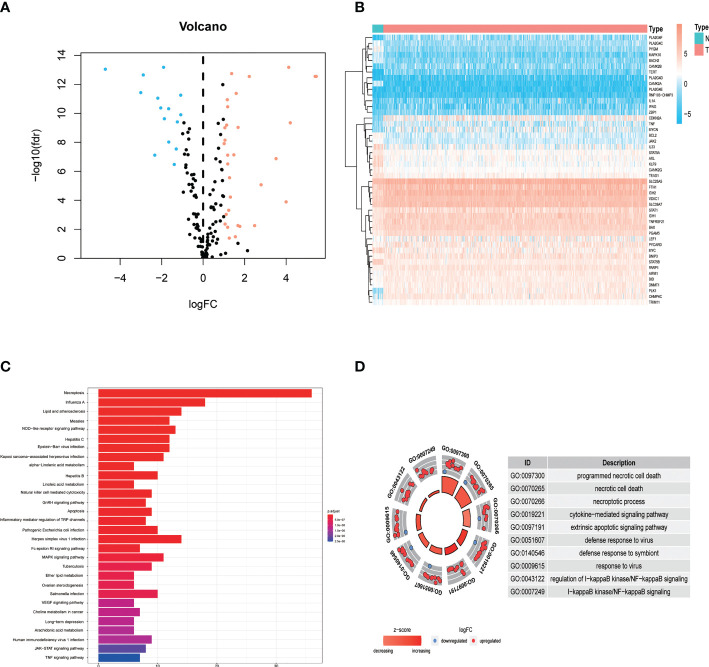
Identification and functional annotation of necroptosis-related differentially expressed genes (DEGs) in patients with EC. **(A)** The volcano plot of 47 necroptosis-related DEGs in EC, blue dots represent downregulated genes and orange dots represent upregulated genes. **(B)** The heatmap shows the expression of necroptosis-related DEGs between normal and cancer tissues. **(C)** KEGG enrichment analysis of necroptosis-related DEGs. **(D)** GO enrichment analysis of necroptosis-related DEGs.

The study design is depicted in **Figure 1**. We obtained 47 necroptosis-related DEGs, consisting of 32 upregulated genes and 15 downregulated genes (**Figure 2A**). A heatmap was created to visualize the expression of necroptosis-related genes between the normal and tumor samples (**Figure 2B**). KEGG enrichment analyses revealed that the selected DEGs were chiefly involved in necroptosis, MAPK signaling pathways, NOD-like receptor signaling pathways, apoptosis, Influenza A, cytotoxicity mediated by natural killer cell, GnRH signaling pathway and lipid and atherosclerosis (**Figure 2C**). Based on the GO analysis of biological process (BP), DEGs were mainly enriched in the programmed necrotic cell death, necrotic cell death, necroptotic process, extrinsic apoptotic signaling pathway and cytokine-mediated signaling pathway. The GO analysis of the cellular components (CC) showed that DEGs were enriched primarily in membrane raft, membrane microdomain, and inflammasome complex. In terms of molecular function (MF), DEGs were enriched primarily in cytokine receptor binding, calcium-dependent phospholipid binding, and phospholipase activity (**Figure 2D**).

### Construction of the necroptosis-related lncRNA predictive signature in EC

**Figure 3:**
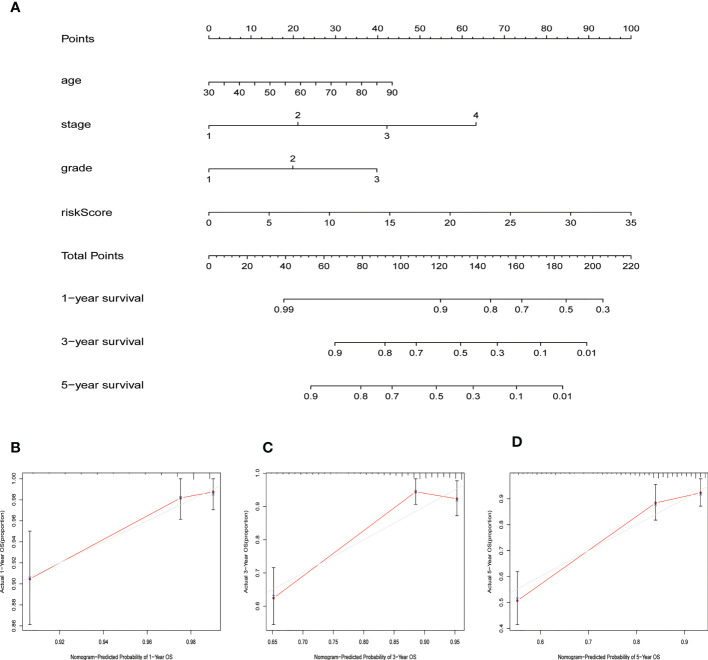
Construction of necroptosis-related lncRNA predictive model and lncRNA-mRNA network of seven necroptosis-related lncRNAs (NRLs). **(A)** Forest plot shows the HR and p value of 48 prognostic-related NRLs from the univariate Cox regression analysis. **(B)** Sankey diagram of prognostic NRLs, consisted of four protective factors and three risk factors. **(C)** Co-expression network for necroptosis-related genes and NRLs. **(D)** The expression profiles of the 7 NRLs in EC samples and normal tissues.

We obtained 539 NRLs from TCGA database, and 48 NRLs that are distinctly correlated with the EC patients prognosis were identified based on univariate Cox regression analysis, (**Figure 3A**). LASSO Cox regression analysis filtered the NRLs (**Supplementary Figure S1**) and multivariate Cox regression analysis ultimately identified 7 NRLs (LEF1-AS1, AC019080.5, AC010503.4, BOLA3-AS1, AC022144.1, AP000345.2, and RPARP-AS1) for developing a predictive signature. These NRLs consisted of four protective factors (LEF1-AS1, AC010503.4, AP000345.2, and RPARP-AS1) and three risk factors (AC019080.5, BOLA3-AS1, and AC022144.1) (**Figures 3B, C**). Based on the transcriptome expression information from the TCGA database, we examined the expression levels of the 7 NRLs in tumor samples and normal tissues (**Figure 3D**). Based on the expression levels and regression coefficients of these 7 lncRNAs, the risk score for EC patients was determined as follows: risk score = (-0.502 × LEF1-AS1 expression) + (0.852 × AC019080.5 expression) + (-0.326 × AC010503.4 expression) + (0.569 × BOLA3-AS1 expression) + (0.644 × AC022144.1 expression) + (-0.842 × AP000345.2 expression) + (-0.714 × RPARP-AS1 expression).

### Assessment and validation of prognostic risk model in EC

**Figure 4:**
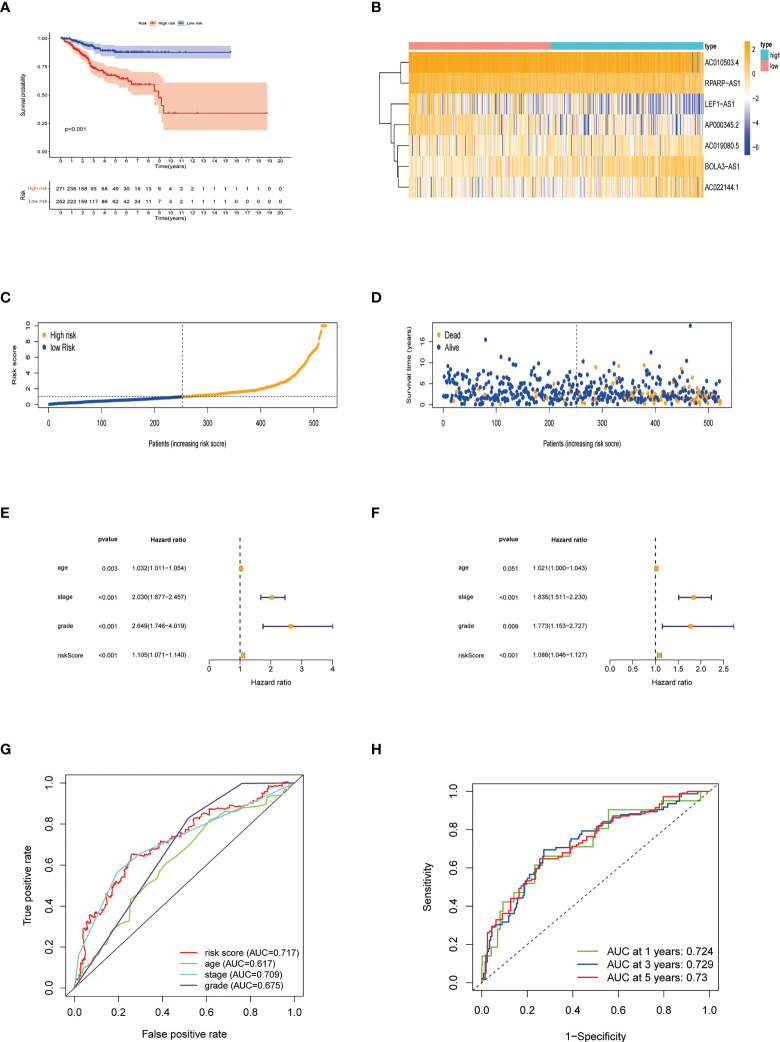
Characteristics of the predictive signature. **(A)** Kaplan-Meier curves for the OS of EC patients in the low- and high-risk groups. **(B)** Expression profiles of 7 necroptosis-related lncRNAs in the low- and high-risk groups. **(C)** The risk score distribution between these two groups. The black dotted line is the optimal cut-of value for dividing patients into low- and high-risk groups. **(D)** The distribution of survival status of patients with different risk scores. Blue dots represent the number of survivors, and orange dots represent the number of deaths. **(E)** Forest plot for univariate Cox regression analysis of clinical factors and risk score with OS. **(F)** Forest plot for multivariate Cox regression analysis of clinical factors and risk score with OS. The necroptosis-related lncRNAs signature is an independent prognostic factor. **(G)** The ROC curve of the risk score and other clinicopathological variables. **(H)** The ROC curve and AUC values at 1-, 3-, and 5-year survival for the predictive signature.

**Figure 5:**
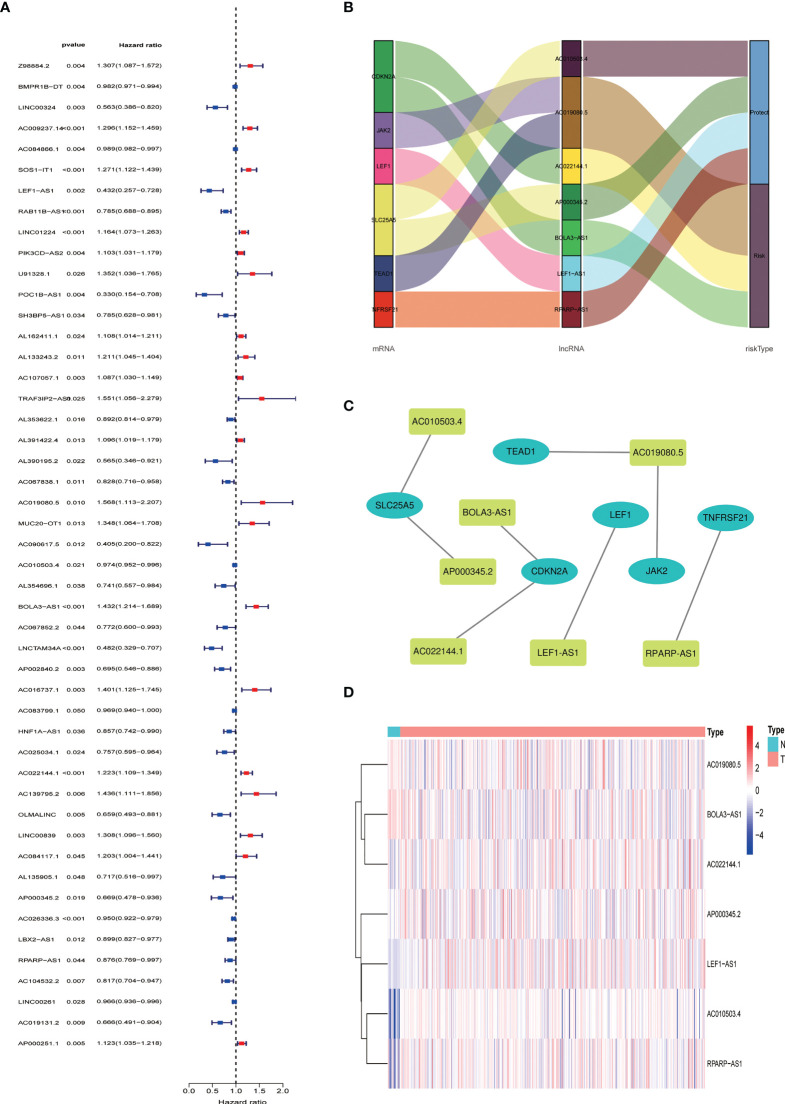
Nomogram for predicting overall survival (OS). **(A)** A prognostic nomogram combining risk score with clinicopathological variables for predicting 1-, 3-, and 5-year OS in EC patients. **(B–D)** The calibration curves of the nomogram confirming consistency between the predicted 1-, 3-, and 5-year survival rates and the actual OS probabilities.

Patients were divided into low- and high-risk groups based on the median value of risk score. Kaplan-Meier analyses revealed that patients in the high-risk cohort had shorter OS times (**Figure 4A**, all p<0.001). **Figure 4B** shows the expression of the 7 NRLs in the low- and high-risk groups, and **Figure 4C** shows the risk scores of these two groups. As risk scores increased, the poorer the patient prognosis was (**Figure 4D**). Using univariate Cox regression analysis, we observed that stage, age, risk score, and grade were correlated with the OS of patients with EC (**Figure 4E**). Multivariate Cox regression analysis revealed that risk score, stage, and grade were independent predictors for patients with EC (**Figure 4F**). The AUC of the risk score can be used to better predict the patients’ prognosis than other clinicopathological variables based on the result of multivariate cox regression (**Figure 4G**). The AUC values of 1-, 3-, and 5-year survival were 0.724, 0.729, and 0.730, respectively, suggesting that the NRLs signature was able to strongly predict the outcome in EC patients (**Figure 4H**). The calibration curves of the nomogram confirmed that the actual OS probabilities were almost consistent with the predicted 1-, 3-, and 5-year survival rates (**Figures 5A–D**).

### Principal component analysis

**Figure 6:**
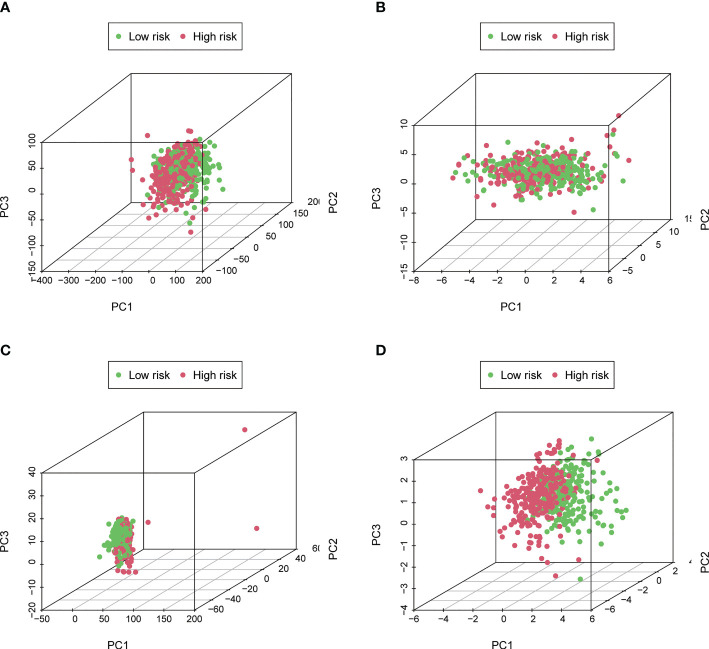
Principal Component Analysis. **(A–D)** The PCA 3D scatterplot of sample distribution based on entire genes, necroptosis-related genes, NRLs, and the risk score, respectively. The risk score has the best discrimination ability to distinguish between the two groups.

The expression levels of different gene types (entire genes, necroptosis-related genes, and NRLs) in high- and low-risk groups were exhibited in **Figure 6**. PCA results revealed that the risk model consisting of 7 NRLs had the best discrimination ability to distinguish between the two groups (**Figure 6D**).

### Stratified prognostic analysis and correlation between the predictive signature and clinicopathological features

**Figure 7:**
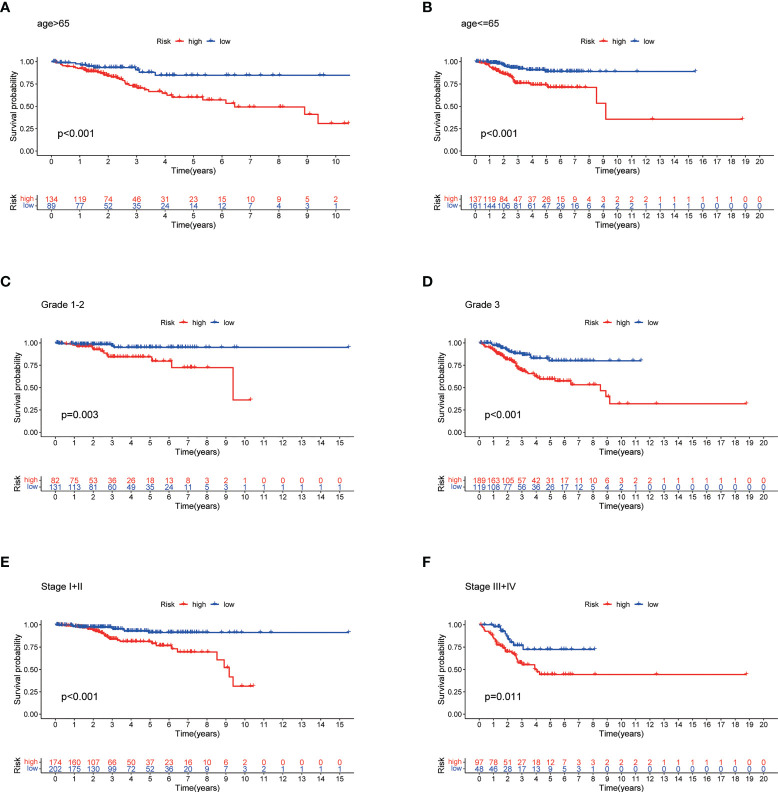
Stratified Prognostic Analysis. Kaplan-Meier survival curves of OS for patients in the low- and high-risk groups stratified by different clinicopathological variables. **(A)** Patients with age >65. **(B)** Patients with age ≤65. **(C)** Patients with grades 1-2. **(D)** Patients with grade 3. **(E)** Patients with stages I-II. **(F)** Patients with stages III-IV. P<0.05.

The high-risk group patients had distinctly poorer OS across all subgroups of age (>65 and ≤65 years old), stage (I-II and III-IV), and grade (G1-2 and G3) (**Figures 7A–F**). These results indicate the high reliability of the risk model. In addition, we investigated the expression distribution of the 7 prognostic NRLs and the clinicopathological parameters in the low- and high-risk groups, and the results are shown in a heat map (**Supplementary Figure S2**).

### Internal verification of the predictive signature

To explore the predictive ability of the predictive risk model for the prognosis of EC, we randomly divided patients with EC into training cohort and testing cohort. In both training and testing sets, the high-risk group patients had a more adverse OS compared with low-risk group patients (**Supplementary Figures S3A, B**). The expression heatmaps of the 7 NRLs in the two groups are shown in **Supplementary Figures S3C, D**, and the risk score distribution and the survival status in the training and testing sets are showed in **Supplementary Figures S3E–H**. In the training cohort, the AUC values of 1-, 3-, and 5-year OS rates were 0.757, 0.760, and 0.738, respectively (**Supplementary Figure S3I**), while in the testing cohort, the AUC values of 1-, 3-, and 5-year OS rates were 0.687, 0.690, and 0.716, respectively (**Supplementary Figure S3J**). The ROC curves of the two internal sets indicate the good predictive capability of the risk model.

### Correlation between risk score and seven prognostic lncRNAs as well as clinicopathological variables

**Figure 8:**
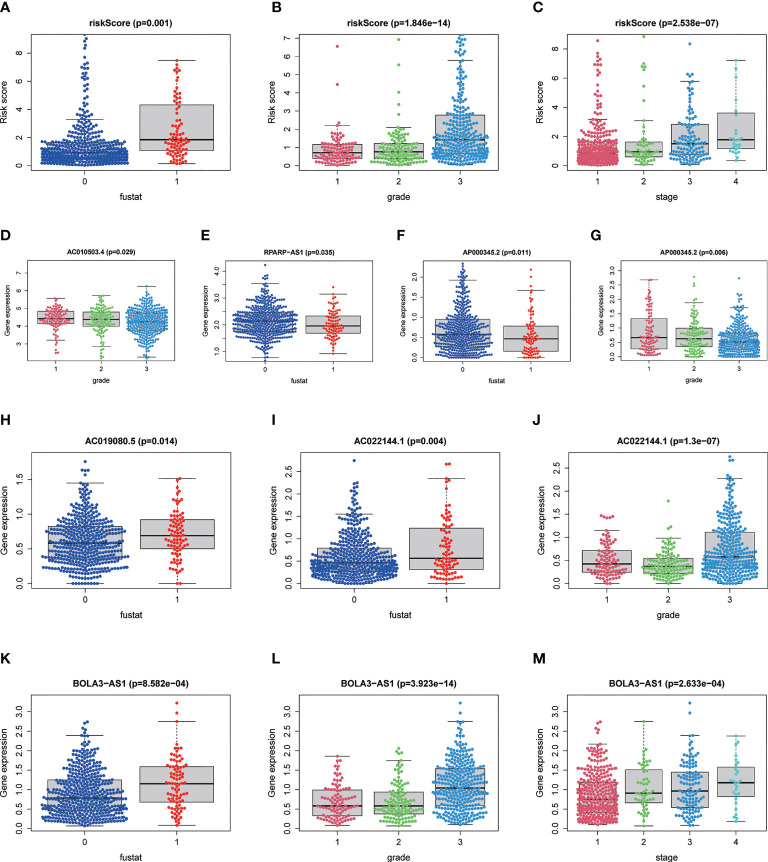
Correlation between risk score and seven prognostic lncRNAs as well as clinicopathological variables. **(A–C)** Risk scores correlated with survival status, grade, and stage. **(D)** Expression level of AC010503.4 correlated with grade. **(E)** Expression level of RPARP-AS1 correlated with survival status. **(F, G)** Expression level of AP000345.2 correlated with grade and survival status. **(H)** Expression level of AC019080.5 correlated with survival status. **(I, J)** Expression level of AC022144.1 correlated with grade and survival status. **(K–M)** Expression level of BOLA3-AS1 associated with grade, stage, and survival status, respectively. P<0.05.

Our results showed that risk score was significantly associated with patient survival status, grade, and stage (**Figures 8A–C**). Among the 7 NRLs, three protective NRLs and three risk NRLs were found to be distinctly correlated with clinicopathological variables. AC010503.4 was associated with grade (**Figure 8D**), RPARP-AS1 was associated with fustat (**Figure 8E**), and AP000345.2 was correlated with grade and fustat (**Figures 8F, G**). AC019080.5 was associated with fustat (**Figure 8H**), AC022144.1 was correlated with grade and fustat (**Figures 8I, J**), and BOLA3-AS1 was associated with grade, stage, and fustat (**Figures 8K–M**).

### Gene set enrichment analysis between low- and high-risk cohorts

**Figure 9:**
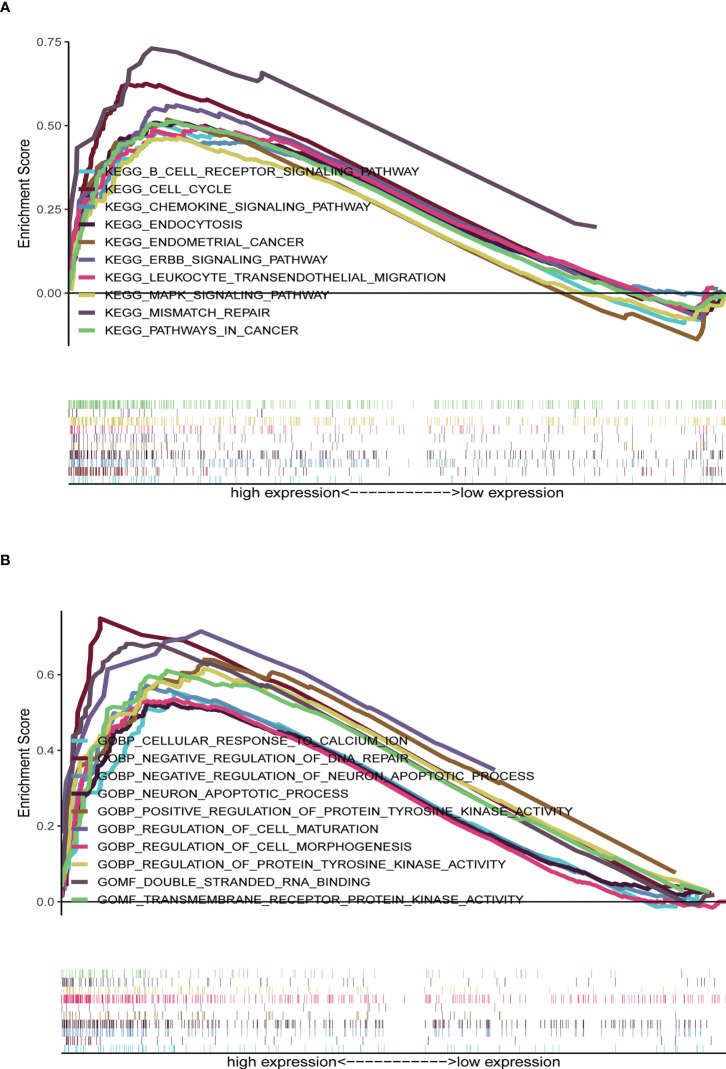
Gene set enrichment analysis (GSEA) between high- and low-risk groups. **(A)** KEGG enrichment analysis of the high- and low-risk groups. **(B)** GO enrichment analysis of the high- and low-risk groups.

KEGG analyses showed that EC, pathways in cancer, the ERBB signaling pathway, the MAPK signaling pathway, the chemokine signaling pathway, the B cell receptor signaling pathway, endocytosis, cell cycles, mismatch repair, and leukocyte transendotheliat migration were significantly enriched in the high-risk group (**Figure 9A**, **Table 1**). GO enrichment analyses indicated that biological functions including transmembrane receptor protein kinase activity, negative regulation of DNA repair, positive regulation of protein tyrosine kinase activity, negative regulation of neuron apoptotic process, neuron apoptotic process, cellular response to calcium ion, regulation of protein tyrosine kinase activity, regulation of cell maturation, regulation of cell morphogenesis involved in differentiation, and double stranded RNA binding were enriched in the high-risk group (**Figure 9B**, **Table 2**).

**Table 1:** The high-risk group enriched gene sets.

Gene Set	ES	NES	NOM p-val	FDR q-val
Endometrial cancer	0.52	1.68	0.026	0.063
Pathways in cancer	0.51	2.01	0.000	0.027
MAPK signaling pathway	0.46	1.88	0.000	0.023
ERBB signaling pathway	0.56	1.98	0.000	0.021
Endocytosis	0.51	1.93	0.002	0.019
Cell cycle	0.63	1.88	0.007	0.024
Mismatch repair	0.73	1.70	0.019	0.057
Leukocyte transendotheliat migration	0.50	1.79	0.012	0.041
Chemokine signaling pathway	0.48	1.67	0.035	0.064
B cell receptor signaling pathway	0.51	1.66	0.042	0.065

ES, enrichment score; NES, normalized enrichment score; NOM, nominal; FDR, false discovery rate; MAPK, mitogen-activated protein kinase.

**Table 2:** The high-risk group enriched gene sets by GO analyses.

Gene Set	ES	NES	NOM p-val	FDR q-val
Cellular response to calcium ion	0.55	2.13	0.000	0.017
Negative regulation of DNA repair	0.75	2.17	0.000	0.019
Negative regulation of neuron apoptotic process	0.57	2.21	0.000	0.012
Neuron apoptotic process	0.52	2.13	0.000	0.017
Positive regulation of protein tyrosine kinase activity	0.64	2.24	0.000	0.010
Regulation of cell maturation	0.71	2.15	0.000	0.018
Regulation of cell morphogenesis involved in differentiation	0.58	2.12	0.000	0.016
Regulation of protein tyrosine kinase activity	0.62	2.21	0.000	0.013
Double stranded RNA binging	0.68	2.16	0.000	0.020
Transmembrane receptor protein kinase activity	0.61	2.14	0.000	0.017

ES, enrichment score; NES, normalized enrichment score; NOM, nominal; FDR, false discovery rate.

### Analysis of tumor infiltrating immune cell and immune function

**Figure 10:**
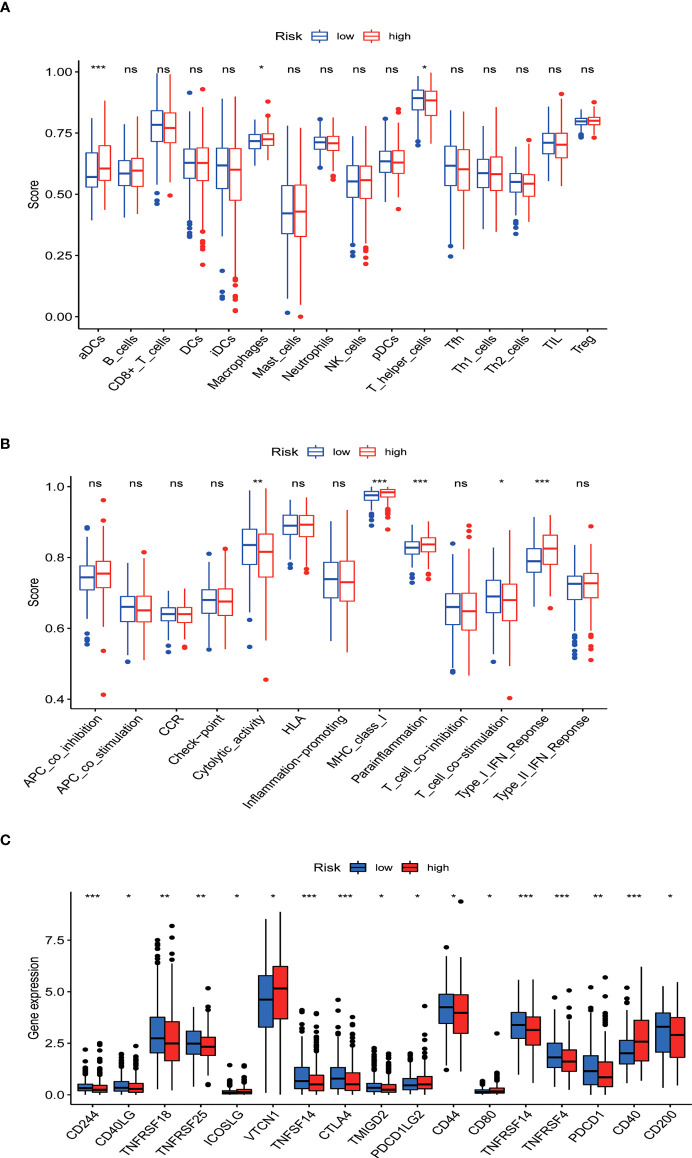
Tumor infiltrating immune cell and immune function. **(A)** Comparison between the immune cell infiltration levels in low- and high-risk groups. **(B)** Correlations between predictive signature and immune-related functions. **(C)** Differential expression of immune checkpoints in patients between low- and high-risk groups. ^*^P<0.05, ^**^P<0.01, and ^***^P<0.001. ns: not significant.

ssGSEA results revealed that different tumor infiltrating immune cells differed significantly between the low- and high-risk groups. The high-risk group had higher levels of activated dendritic cells (aDCs) and macrophages, while the low-risk group had a higher level of T helper cells (**Figure 10A**). We also found that the high-risk group had higher type I IFN response, immune function scores of MHC-class I, and parainflammation, while the low-risk group had higher T cell co-stimulation and immune function scores of cytolytic activity (**Figure 10B**). Furthermore, the expression levels of immune checkpoints (CD244, CD40LG, TNFRSF18, TNFRSF25, TNFSF14, CTLA4, TMIGD2, CD44, TNFRSF14, TNFRSF4, PDCD1, and CD200) were lower in the high-risk group (**Figures 10C**).

### Relationship between the predictive signature and drug sensitivity in EC therapy

**Figure 11:**
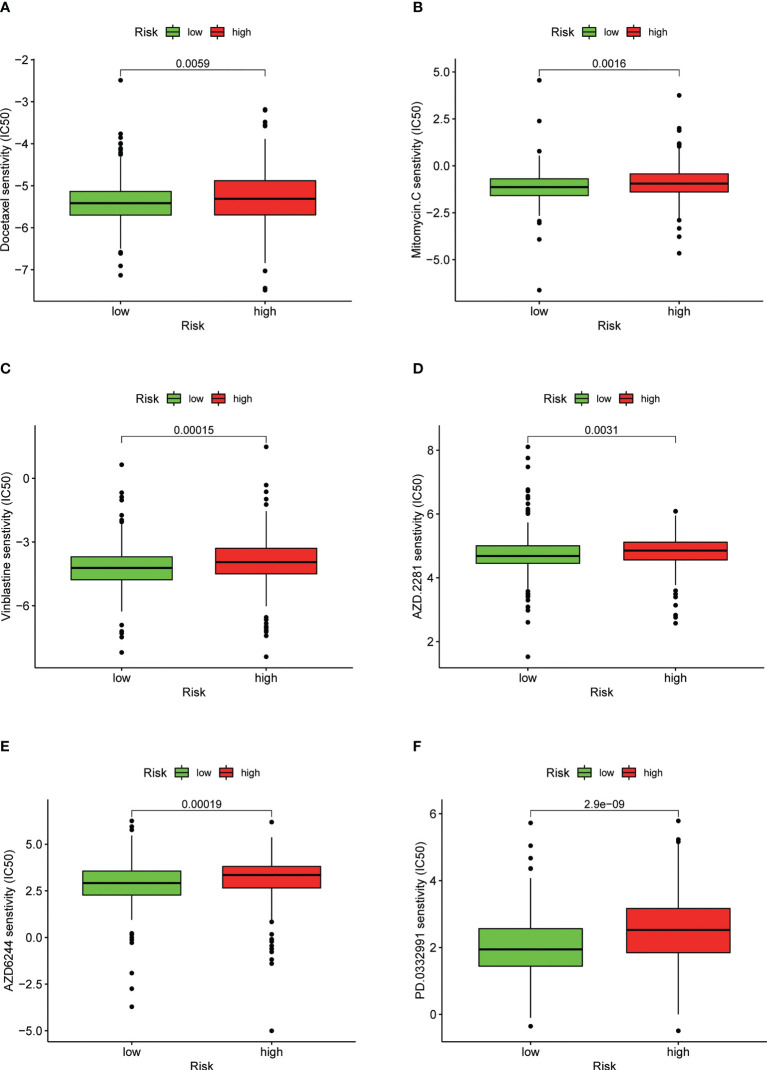
Drug sensitivity between low- and high-risk groups. **(A)** IC50 of docetaxel in low- and high-risk groups. **(B)** IC50 of mitomycin.C in low- and high-risk groups. **(C)** IC50 of vinblastine in low- and high-risk groups. **(D)** IC50 of AZD.2281 (olaparib) in low- and high-risk groups. **(E)** IC50 of AZD6244 in low- and high-risk groups. **(F)** IC50 of PD.0332991 (palbociclib) in low- and high-risk groups.

We found that the IC50 values of docetaxel, mitomycin.C, vinblastine, AZD.2281 (olaparib), AZD6244, PD.0332991 (palbociclib) (**Figures 11A–F**), BIBW2992 (afatinib), vorinostat, NVP.BEZ235, AUY922 (NVP-AUY922), camptothecin, metformin, MG.132, cyclopamine, and MK.2206 were higher in the high-risk group than in the low-risk group (**Supplementary Figures S4A–J**). In contrast, the IC50 values of pazopanib, AP.24534 (ponatinib), AZD.0530 (saracatinib), AZD6482, ABT.263 (navitoclax), Imatinib, PF.02341066 (crizotinib), ATRA, and sorafenib were lower in the high-risk group than in the low-risk group (**Supplementary Figures S5A–I**).

## Discussion

Despite the progress made in EC treatment, the response rates remain insufficient. While some studies have suggested that necroptosis plays a crucial role in the progression, metastasis, and prognosis of cancer (8–11), others have observed that necroptosis prevents tumor progression (12, 13). In addition, increasing evidence suggests that the prognosis of cancer patients can be predicted by constructing necroptosis-related lncRNA predictive signatures. Whether necroptosis-related lncRNA signature can predict the prognosis and anticancer sensitivity in treatment of EC patients needs to be illuminated

Here, we acquired 47 necroptosis-related DEGs. Subsequent KEGG enrichment analyses revealed that these DEGs were mainly involved in necroptosis, lipid and atherosclerosis, the NOD-like receptor signaling pathway, GnRH signaling pathway, natural killer cell mediated cytotoxicity, the MAPK signaling pathway, apoptosis, and Influenza A. Previous study showed that the upregulated expression of the NOD-like receptor family NLRP3 inflammasome plays a critical role in neuronal necroptosis (32). Polystyrene nanoplastics or lipopolysaccharide have been shown to be able to induce oxidative stress and then activate the MAPK signaling pathway, eventually resulting in necroptosis and inflammation in mice spleens (33). These findings indicate that necroptosis is related with the MAPK signaling pathway and NOD-like receptor signaling pathway.

Previous studies showed that lncRNAs play a critical role in EC (25–29). Here, we acquired 48 NRLs distinctly related to the prognosis of EC patients and identified 7 NRLs for constructing the risk model. Based on the regression coefficient and expression levels of these NRLs, we determined a ‘risk score’, which was used to divide the EC patients into low- and high-risk groups. The high-risk group patients had a shorter survival than low-risk group patients. The ROC curve reveals that the risk model has a precise predictive performance. The predictive signature was even more precise when compared with other clinical parameters; moreover, this signature can predict the prognosis independently without considering other clinicopathological variables. Subsequently, the nomogram and the calibration curves reveal that the actual OS probabilities have an excellent concordance with the prediction. Moreover, we found a distinct discrimination ability between the high- and low-risk groups in the risk model consisting of 7 NRLs. Finally, patients in the high-risk group were found to have distinctly poor OS across age, grade, and stage. Taken together, these results indicate the high reliability of the risk model. We also verified the accuracy of the predictive signature by testing internal cohorts for EC and the observed results are consistent with those of the larger population.

We found that these NRLs were mainly involved in EC, B cell receptor signaling pathway, pathways in cancer, the ERBB signaling pathway, cell cycle, mismatch repair, the chemokine signaling pathway, and the MAPK signaling pathway. Other studies have also found that lncRNA HEIH regulates paclitaxel-resistance, viability and proliferation of endometrial cancer cells through MAPK signaling pathway (34). Arsenic trioxide regulates mRNA and protein expression of estrogen receptor-alpha in endometrial cancer cells through interaction with the MAPK pathway (35). E2 induces telomerase activity through MAPK signaling pathway in endometrial cancer cells (36). There is increasing evidence that the B cell receptor signaling pathway plays an important role in lymphocytic survival, differentiation, proliferation, and trafficking and can promote the survival and growth of malignant B cells by regulating BCR receptor pathway in B cell lymphoma (37). These findings suggest that high-risk group patients were strongly affected by tumor- and immune-related signaling pathways.

aDCs and macrophages were found to have a higher level in the high-risk group than in the low-risk group. Prior research has indicated that immune-related gene USP18 can be regulated by LHX2, affecting aDCs and the MAPK pathway to promote the development of extranodal diffuse large B cell lymphoma (38). Tumor-associated macrophages can enhance the invasion and extravasation of tumor cells and inhibit antigen presentation to further support tumor metastasis (38). In addition, the immune function scores of MHC-class I, parainflammation, and type I IFN response - which are associated with decreased antitumor immunity - were higher in the high-risk group than in the low-risk group. Therefore, the suppressed antitumor immunity in the high-risk group may result in an adverse prognosis. Additionally, we found that the expression levels of immune checkpoints (CD244, CD40LG, TNFRSF18, TNFRSF25, TNFSF14, CTLA4, TMIGD2, CD44, TNFRSF14, TNFRSF4, PDCD1, and CD200) were consistently lower in the high-risk group than in the low-risk group, meaning that the high-risk group was a failure sub-type for treatment using immune-checkpoint inhibitors. These results are helpful for understanding the lack of effectiveness of immune-checkpoint inhibitor treatment for EC patients. Our results indicated that high-risk patients are likely sensitive to conventional chemotherapeutic agents and common targeted drugs, such as Docetaxel, Mitomycin.C, Vinblastine, AZD.2281 (olaparib), AZD6244, PD.0332991 (Palbociclib), BIBW2992 (afatinib), Vorinostat, NVP.BEZ235, AUY922 (NVP-AUY922), Camptothecin, Metformin, MG.132, Cyclopamine, and MK.2206. In contrast, these patients were resistant to Pazopanib, AP.24534 (ponatinib), AZD.0530 (saracatinib), AZD6482, ABT.263 (navitoclax), Imatinib, PF.02341066 (crizotinib), ATRA, and Sorafenib. Taken together, these findings allow us to better choose individualized treatment options for patients.

We note that this study had certain limitations. Firstly, due to the limitations of using historic databases, our study lacked external validation of the applicability of the predictive signature. Secondly, data from multicenter clinical cohorts are also needed to verify the predictive signature. Finally, the underlying mechanisms of these NRLs in EC remain to be further investigated. Some functional experiments are needed to validate our findings and reveal the detailed molecular mechanisms of these NRLs in EC.

## Conclusion

We identified a novel necroptosis-related lncRNA signature to predict the prognosis of EC patients, which might provide a novel therapeutic strategy for EC. This work may help to choose and develop individualized and precise treatment options for patients with EC.

## Data availability statement

The raw data supporting the conclusions of this article will be made available by the authors, without undue reservation.

## Ethics statement

Ethical review and approval was not required for the study on human participants in accordance with the local legislation and institutional requirements. Written informed consent for participation was not required for this study in accordance with the national legislation and the institutional requirements.

## Author contributions

W-PH conducted research design, data analysis, and drafted the manuscript. Y-YC performed the literature search and helped to draft the manuscript. L-XW performed the literature search and helped to edit the manuscript. Y-YG helped with editing and collating references. Z-SY coordinated and evaluated the literature review. G-FY reviewed and revised the manuscript. All authors contributed to the article and approved the submitted version.

## Funding

This study was supported by grants from the Nature Science Foundation of China (No.81772769), the Research Project in the Science and Technology Bureau in Guangzhou (No. 201704020125), and the Guangdong Basic and Applied Basic Research Foundation (No. 2020A1515010169).

## Conflict of interest

The authors declare that the research was conducted in the absence of any commercial or financial relationships that could be construed as a potential conflict of interest.

The handling editor HW declared a shared affiliation with the authors at the time of review.

## Publisher’s note

All claims expressed in this article are solely those of the authors and do not necessarily represent those of their affiliated organizations, or those of the publisher, the editors and the reviewers. Any product that may be evaluated in this article, or claim that may be made by its manufacturer, is not guaranteed or endorsed by the publisher.
