# Supplementary material for: A novel necroptosis-related lncRNA signature for predicting prognosis and anti-cancer treatment response in endometrial cancer
Source: Front Immunol. 2022 Nov 16;13:1018544. doi: 10.3389/fimmu.2022.1018544 (PMC9708746; doi:10.3389/fimmu.2022.1018544)
Supplement: Supplementary file 1 [file DataSheet_1.pdf]

Figure S1

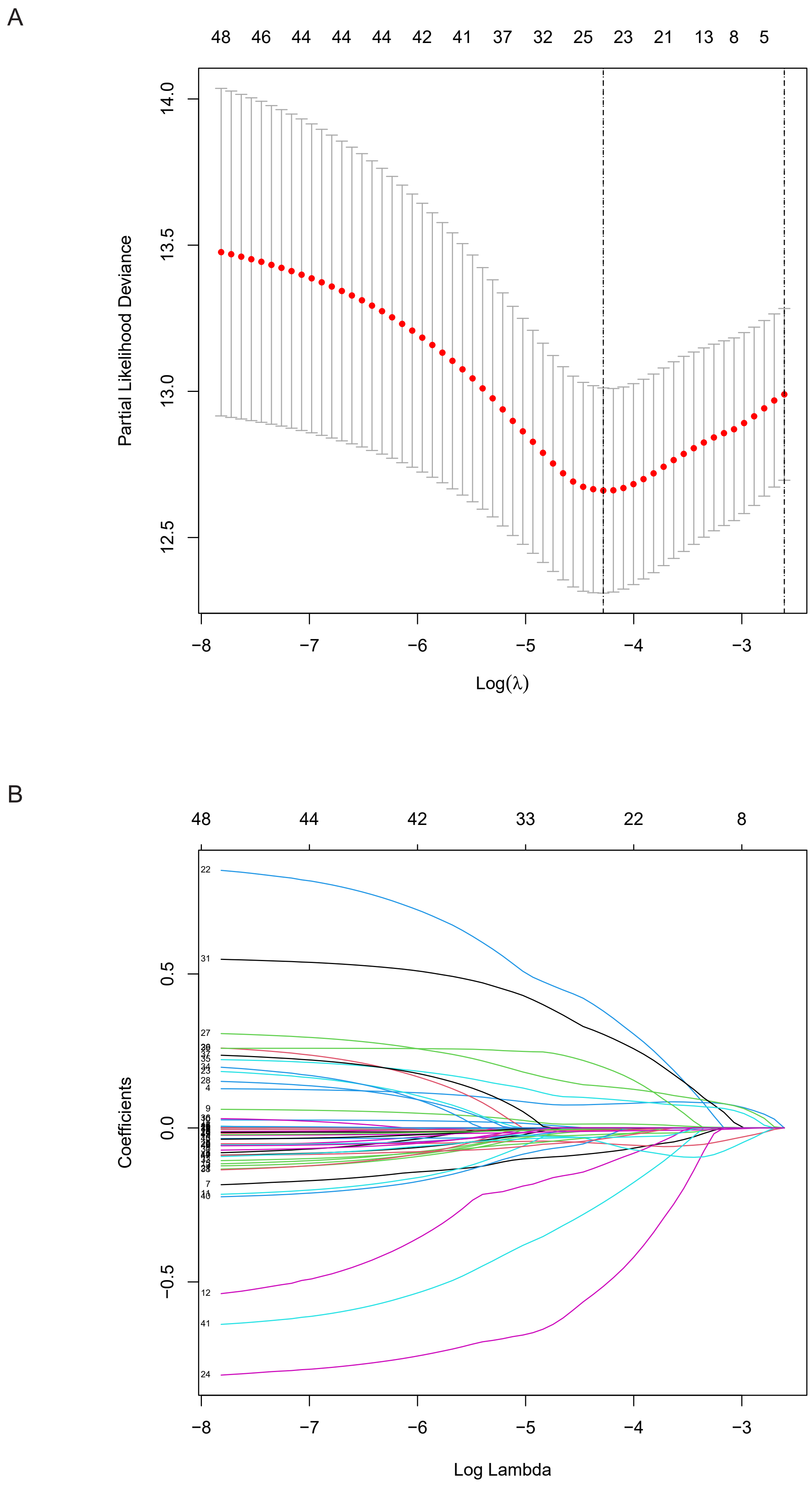
A
48 46 44 44 44 42 41 37 32 25 23 21 13 8 5
Partial Likelihood Deviance
14.0
13.5
13.0
12.5
−8 −7 −6 −5 −4 −3
Log(λ)
B
48 44 42 33 22 8
Coefficients
0.5
0.0
−0.5
−8 −7 −6 −5 −4 −3
Log Lambda

Figure S2

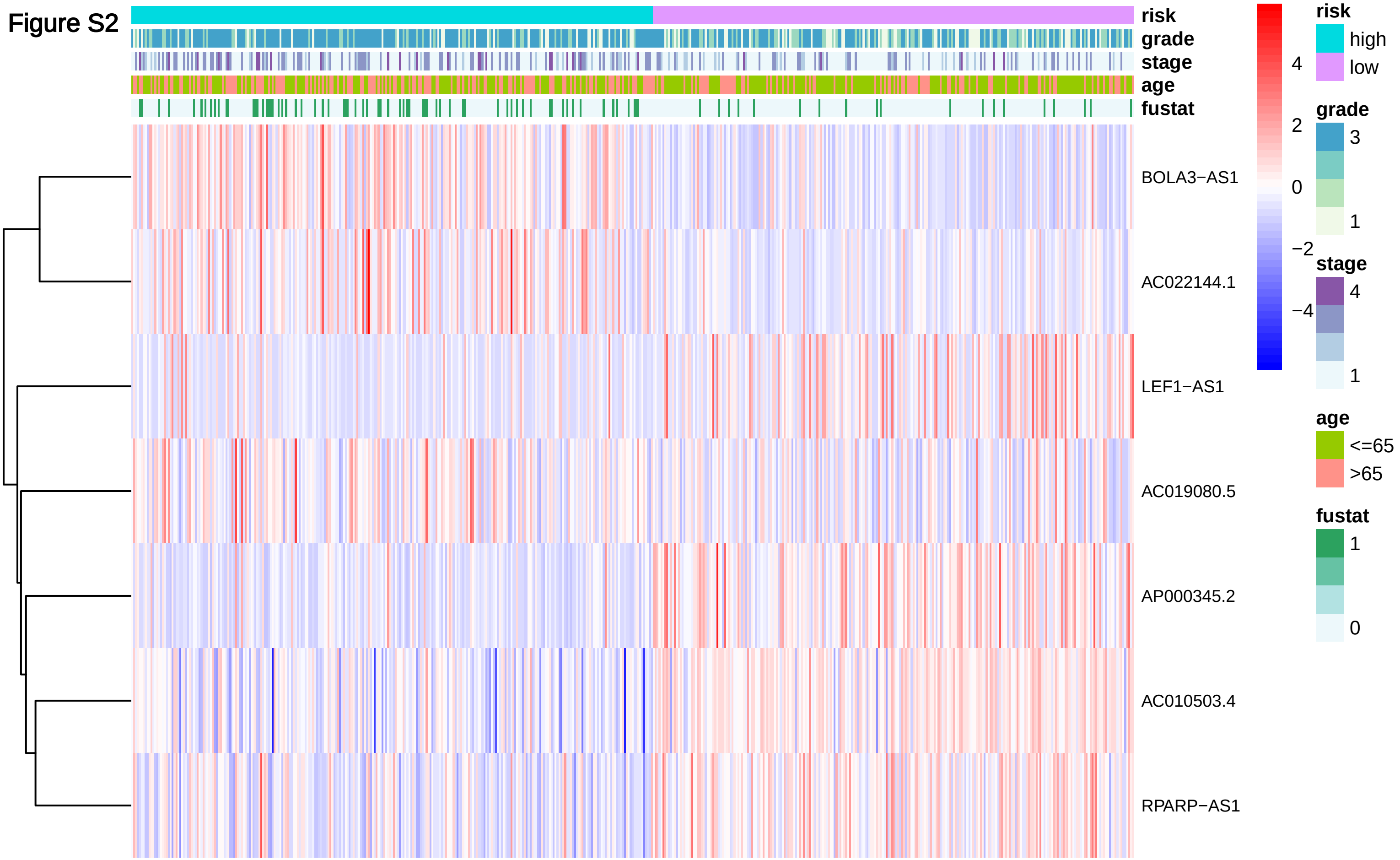
risk
grade
stage
age
fustat
BOLA3−AS1
AC022144.1
LEF1−AS1
AC019080.5
AP000345.2
AC010503.4
RPARP−AS1
4
2
0
−2
−4
risk
high
low
grade
3
1
stage
4
1
age
<=65
>65
fustat
1
0

Figure S3

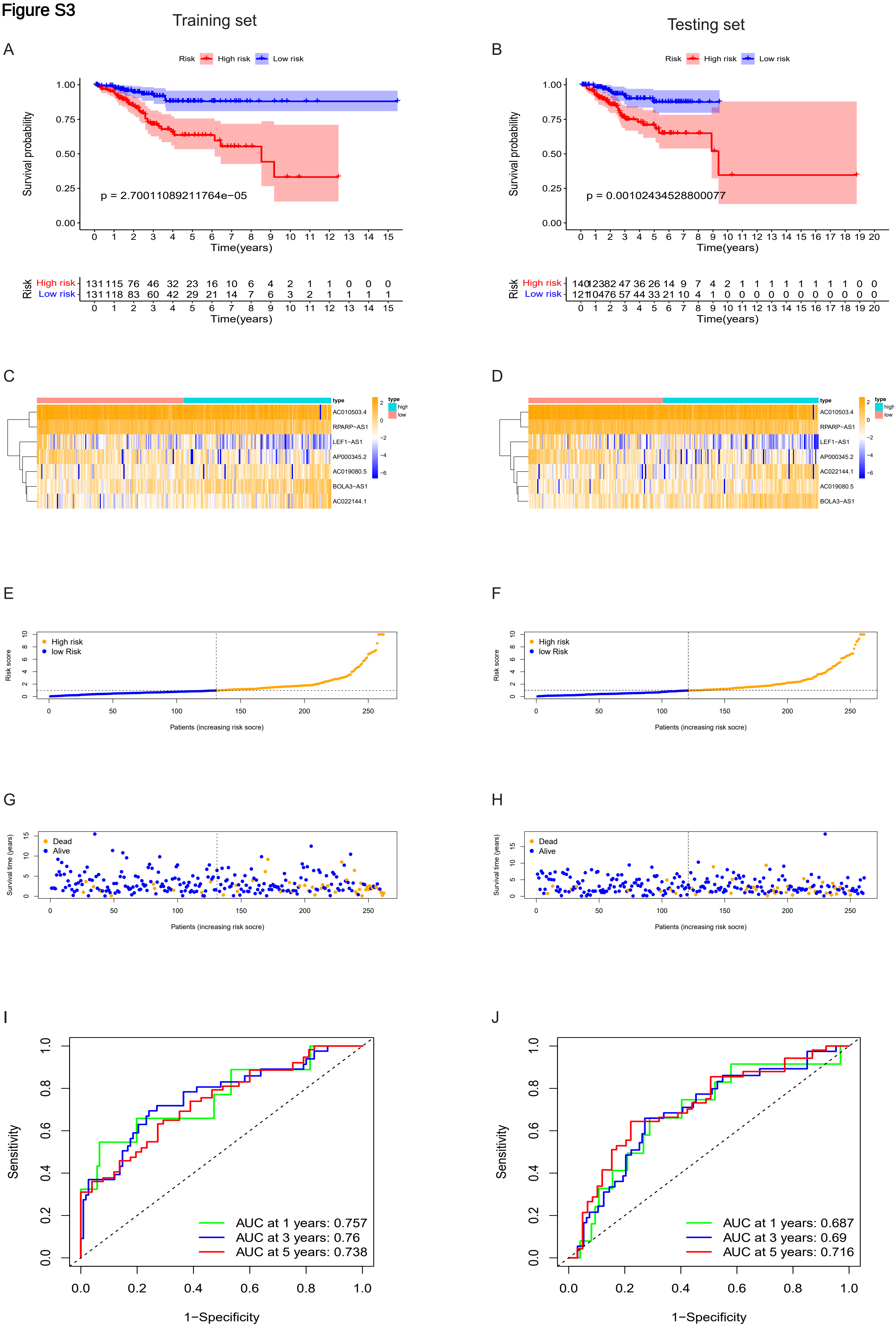
Training set
Testing set
A
B
Risk High risk Low risk
Survival probability
1.00
0.75
0.50
0.25
0.00
p = 2.70011089211764e-05
p = 0.00102434528800077
Time(years)
Risk
High risk
Low risk
131 115 76 46 32 23 16 10 6 4 2 1 1 0 0 0
131 118 83 60 42 29 21 14 7 6 3 2 1 1 1 1
C
D
type
high
low
AC010503.4
RPARP-AS1
LEF1-AS1
AP000345.2
AC019080.5
BOLA3-AS1
AC022144.1
E
F
High risk
low Risk
Risk score
Patients (increasing risk socre)
G
H
Dead
Alive
Survival time (years)
I
J
Sensitivity
1−Specificity
AUC at 1 years: 0.757
AUC at 3 years: 0.76
AUC at 5 years: 0.738
AUC at 1 years: 0.687
AUC at 3 years: 0.69
AUC at 5 years: 0.716

Figure S4

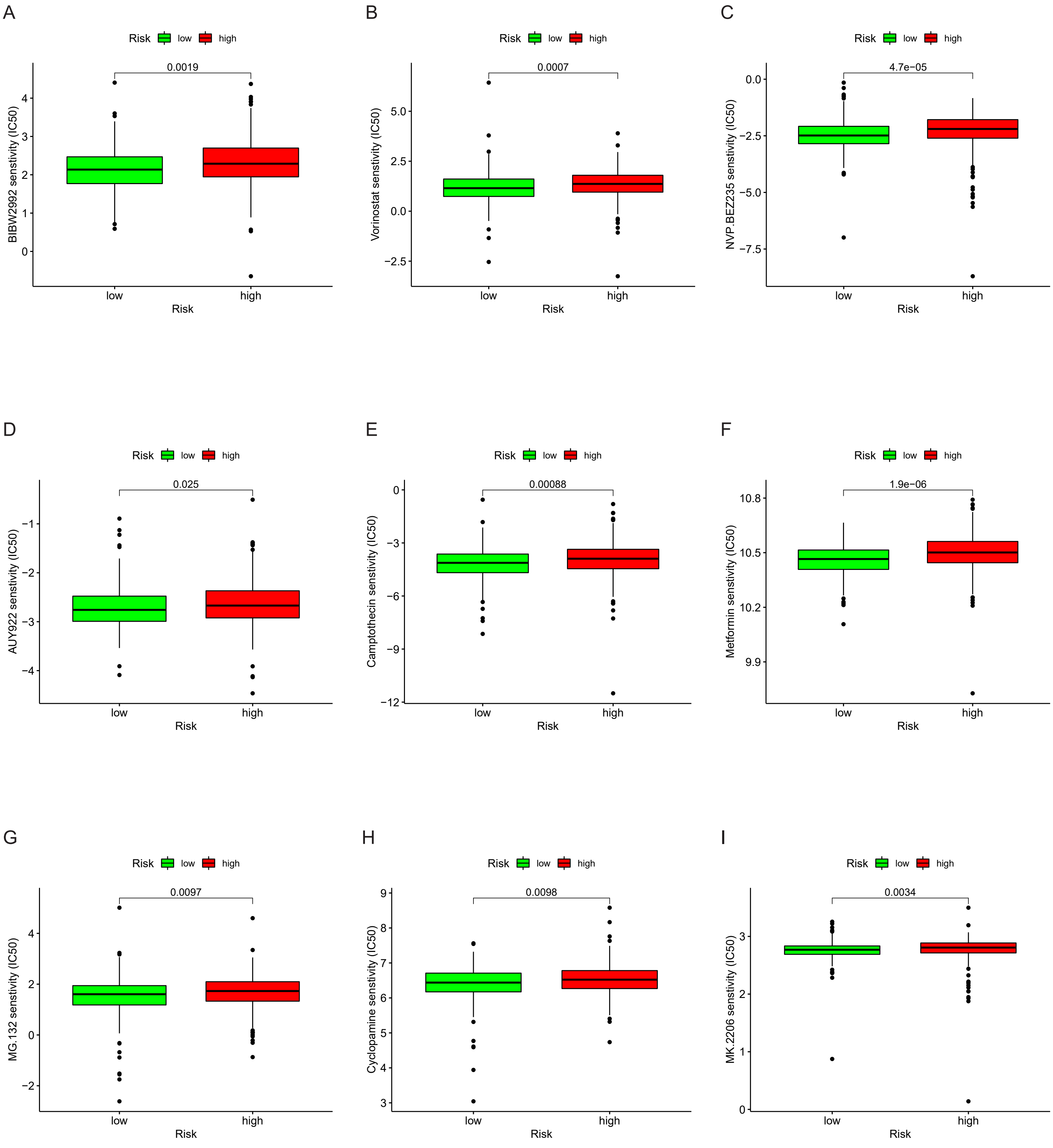
A
Risk low high
0.0019
BIBW2992 sensitivity (IC50)
low high
Risk
B
Risk low high
0.0007
Vorinostat sensitivity (IC50)
low high
Risk
C
Risk low high
4.7e−05
NVP.BEZ235 sensitivity (IC50)
low high
Risk
D
Risk low high
0.025
AUY922 senstivity (IC50)
low high
Risk
E
Risk low high
0.00088
Camptothecin sensitivity (IC50)
low high
Risk
F
Risk low high
1.9e−06
Metformin sensitivity (IC50)
low high
Risk
G
Risk low high
0.0097
MG.132 sensitivity (IC50)
low high
Risk
H
Risk low high
0.0098
Cyclopamine sensitivity (IC50)
low high
Risk
I
Risk low high
0.0034
MK.2206 sensitivity (IC50)
low high
Risk

Figure S5

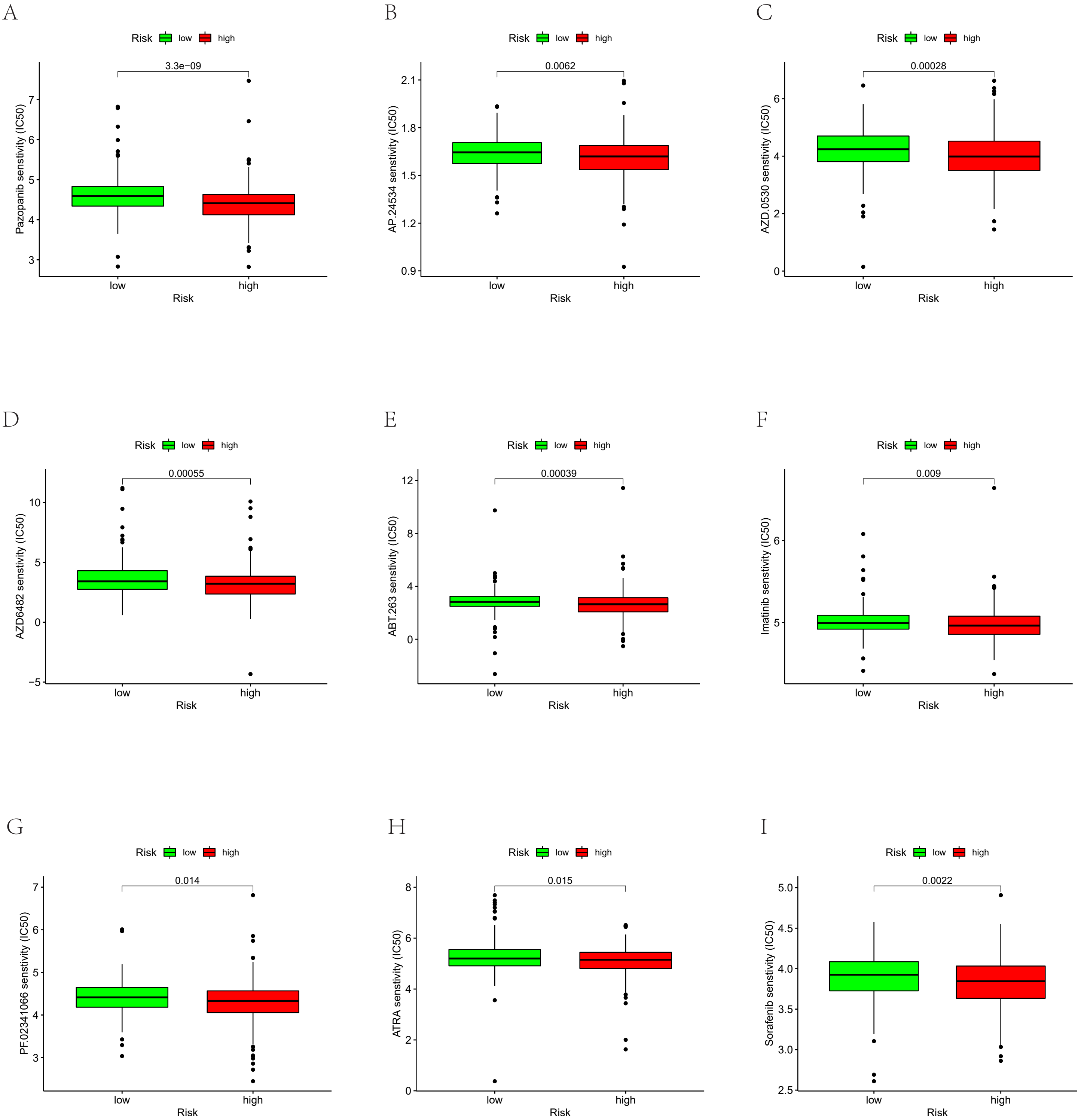

A
Risk low high
3.3e−09
Pazopanib sensitivity (IC50)
low high
Risk
B
Risk low high
0.0062
AP.24534 sensitivity (IC50)
low high
Risk
C
Risk low high
0.00028
AZD.0530 sensitivity (IC50)
low high
Risk
D
Risk low high
0.00055
AZD6482 sensitivity (IC50)
low high
Risk
E
Risk low high
0.00039
ABT.263 sensitivity (IC50)
low high
Risk
F
Risk low high
0.009
Imatinib sensitivity (IC50)
low high
Risk
G
Risk low high
0.014
PF.02341066 sensitivity (IC50)
low high
Risk
H
Risk low high
0.015
ATRA sensitivity (IC50)
low high
Risk
I
Risk low high
0.0022
Sorafenib sensitivity (IC50)
low high
Risk
