# Supplementary material for: A novel necroptosis-related lncRNA signature for predicting prognosis and anti-cancer treatment response in endometrial cancer
Source: Front Immunol. 2022 Nov 16;13:1018544. doi: 10.3389/fimmu.2022.1018544 (PMC9708746; doi:10.3389/fimmu.2022.1018544)
Supplement: Supplementary file 2 [file DataSheet_2.pdf]

Supplementary Figure Legends

Figure S1 LASSO Cox regression model for overall survival.

Figure S2 The heat map of expression distribution of seven prognostic necroptosis-related lncRNAs and clinicopathological variables in the low- and high-risk groups.

Figure S3 Internal verification of the predictive risk model for OS based on the entire cohort. (A-B) Kaplan-Meier curve for the OS of patients with EC in the testing and training sets. (C-D) The expression profile heatmap of 7 necroptosis-related lncRNAs in the testing and training sets. (E-H) The risk score distribution and survival status between low- and high-risk groups in the testing and training sets. (I-J) The ROC curve and AUC values at 1-, 3-, and 5-year survival for the predictive signature in the testing and training sets.

Figure S4 Drug sensitivity between low- and high-risk groups. IC50 values of (A) BIBW2992 (afatinib), (B) vorinostat, (C) NVP.BEZ235, (D) AUY922 (NVP-AUY922), (E) camptothecin, (F) metformin, (G) MG.132, (H) cyclopamine, and (I) MK.2206 were higher in the high-risk group than in the low-risk group.

Figure S5 Comparison of drugs sensitivity between low- and high-risk groups. IC50 values of (A) pazopanib, (B) AP.24534 (ponatinib), (C) AZD.0530 (saracatinib), (D) AZD6482, (E) ABT.263 (navitoclax), (F) Imatinib, (G) PF.02341066 (crizotinib), (H) ATRA, and (I) sorafenib were lower in the high-risk group than in the low-risk group.
