# Supplementary material for: A novel necroptosis-related lncRNA signature for predicting prognosis and anti-cancer treatment response in endometrial cancer
Source: Front Immunol. 2022 Nov 16;13:1018544. doi: 10.3389/fimmu.2022.1018544 (PMC9708746; doi:10.3389/fimmu.2022.1018544)
Supplement: Supplementary file 3 [file Table_1.docx]

Table S1 A list of 178 necroptosis-related genes from the mRNA expression matrix of EC available in TCGA database.

| Gene ID | Gene ID | Gene ID | Gene ID | Gene ID |
| --- | --- | --- | --- | --- |
| AIFM1 | FTL | MAPK9 | TICAM1 | DIABLO |
| ALOX15 | GLUD1 | NLRP3 | TICAM2 | DNMT1 |
| BAX | GLUD2 | PARP1 | TLR3 | BRAF |
| BCL2 | GLUL | PGAM5 | TLR4 | AXL |
| BID | HMGB1 | PLA2G4A | TNF | ID1 |
| BIRC2 | HSP90AA1 | PLA2G4B | TNFAIP3 | CDKN2A |
| BIRC3 | HSP90AB1 | PLA2G4C | TNFRSF10A | HSPA4 |
| CAMK2A | IFNA1 | PLA2G4D | TNFRSF10B | STUB1 |
| CAMK2B | IFNA10 | PLA2G4E | TNFRSF1A | FLT3 |
| CAMK2D | IFNA13 | PLA2G4F | TNFSF10 | HAT1 |
| CAMK2G | IFNA14 | PPIA | TRADD | SIRT2 |
| CAPN1 | IFNA16 | PPID | TRAF2 | SIRT1 |
| CAPN2 | IFNA17 | PYCARD | TRAF5 | PLK1 |
| CASP1 | IFNA2 | PYGB | TRPM7 | MPG |
| CASP8 | IFNA21 | PYGL | TYK2 | BACH2 |
| CFLAR | IFNA4 | PYGM | USP21 | GATA3 |
| CHMP1A | IFNA5 | RBCK1 | VDAC1 | MYCN |
| CHMP1B | IFNA6 | RIPK1 | VDAC2 | ALK |
| CHMP2A | IFNA7 | RIPK3 | VDAC3 | ATRX |
| CHMP2B | IFNA8 | RNF103-CHMP3 | VPS4A | TERT |
| CHMP3 | IFNAR1 | RNF31 | VPS4B | SLC39A7 |
| CHMP4A | IFNAR2 | SHARPIN | XIAP | IDH1 |
| CHMP4B | IFNB1 | SLC25A31 | ZBP1 | IDH2 |
| CHMP4C | IFNG | SLC25A4 | USP22 | KLF9 |
| CHMP5 | IFNGR1 | SLC25A5 | MLKL | HDAC9 |
| CHMP6 | IFNGR2 | SMPD1 | TEAD1 | LEF1 |
| CHMP7 | IL1A | SPATA2 | TSC1 | BNIP3 |
| CYBB | IL1B | SPATA2L | TRIM11 | CD40 |
| CYLD | IL33 | SQSTM1 | IPMK | BCL2L11 |
| DNM1L | IRF9 | STAT1 | ITPK1 | EGFR |
| EIF2AK2 | JAK1 | STAT2 | SIRT3 | DDX58 |
| FADD | JAK2 | STAT3 | MYC | TARDBP |
| FAF1 | JAK3 | STAT4 | TNFRSF1B | APP |
| FAS | JMJD7-PLA2G4B | STAT5A | PANX1 | TNFRSF21 |
| FASLG | MAPK10 | STAT5B | OTULIN |  |
| FTH1 | MAPK8 | STAT6 | MAP3K7 |  |

Table S2 A list of 48 necroptosis-related lncRNAs related to the prognosis of EC patients by univariate Cox regression analysis.

| **gene** | **gene** |
| --- | --- |
| Z98884.2 | AC010503.4 |
| BMPR1B-DT | AL354696.1 |
| LINC00324 | BOLA3-AS1 |
| AC009237.14 | AC067852.2 |
| AC084866.1 | LNCTAM34A |
| SOS1-IT1 | AP002840.2 |
| LEF1-AS1 | AC016737.1 |
| RAB11B-AS1 | AC083799.1 |
| LINC01224 | HNF1A-AS1 |
| PIK3CD-AS2 | AC025034.1 |
| U91328.1 | AC022144.1 |
| POC1B-AS1 | AC139795.2 |
| SH3BP5-AS1 | OLMALINC |
| AL162411.1 | LINC00839 |
| AL133243.2 | AC084117.1 |
| AC107057.1 | AL135905.1 |
| TRAF3IP2-AS1 | AP000345.2 |
| AL353622.1 | AC026336.3 |
| AL391422.4 | LBX2-AS1 |
| AL390195.2 | RPARP-AS1 |
| AC067838.1 | AC104532.2 |
| AC019080.5 | LINC00261 |
| MUC20-OT1 | AC019131.2 |
| AC090617.5 | AP000251.1 |

| **gene** | **coef** | **HR** | **HR.95L** | **HR.95H** | **pvalue** |
| --- | --- | --- | --- | --- | --- |
| LEF1-AS1 | -0.502495693 | 0.605018833 | 0.287952369 | 1.271209504 | 0.184676679 |
| AC019080.5 | 0.85182748 | 2.34392642 | 1.141846133 | 4.811498592 | 0.020261956 |
| AC010503.4 | -0.326132229 | 0.72170975 | 0.512229229 | 1.01685912 | 0.062267039 |
| BOLA3-AS1 | 0.569441313 | 1.76727942 | 1.012555211 | 3.084549378 | 0.045083895 |
| AC022144.1 | 0.64430443 | 1.904661743 | 1.153158853 | 3.14591207 | 0.011850329 |
| AP000345.2 | -0.842095394 | 0.430806867 | 0.203956638 | 0.90997066 | 0.027296598 |
| RPARP-AS1 | -0.713677784 | 0.489839357 | 0.256876281 | 0.93407844 | 0.030232343 |

Table S3 Mutivariate cox proportional hazard regression analysis results of necroptosis-related lncRNAs in EC.
